# Prostate cancer diagnosis using epigenetic biomarkers, 3D high-content imaging and probabilistic cell-by-cell classifiers

**DOI:** 10.18632/oncotarget.18985

**Published:** 2017-07-05

**Authors:** Darko Stefanovski, George Tang, Kolja Wawrowsky, Raymond C. Boston, Nils Lambrecht, Jian Tajbakhsh

**Affiliations:** ^1^ Department of Clinical Studies, School of Veterinary Medicine, University of Pennsylvania, Philadelphia, PA, USA; ^2^ Translational Cytomics Group, Department of Surgery, Cedars-Sinai Medical Center, Los Angeles, CA, USA; ^3^ Department of Biomedical Sciences, Cedars-Sinai Medical Center, Los Angeles, CA, USA; ^4^ Pathology and Laboratory Medicine Service, Veterans Affairs Medical Center, Long Beach, CA, USA; ^5^ Department of Pathology and Laboratory Medicine, University of California Irvine, Orange, CA, USA; ^6^ Samuel Oschin Comprehensive Cancer Institute, Cedars-Sinai Medical Center, Los Angeles, CA, USA

**Keywords:** prostate cancer, epigenetics, 3D high-content imaging, tissue diagnostics, cell heterogeneity

## Abstract

**Background:**

Prostate cancer (PCa) management can benefit from novel concepts/biomarkers for reducing the current 20-30% chance of false-negative diagnosis with standard histopathology of biopsied tissue.

**Method:**

We explored the potential of selected epigenetic markers in combination with validated histopathological markers, 3D high-content imaging, cell-by-cell analysis, and probabilistic classification in generating novel detailed maps of biomarker heterogeneity in patient tissues, and PCa diagnosis. We used consecutive biopsies/radical prostatectomies from five patients for building a database of ∼140,000 analyzed cells across all tissue compartments and for model development; and from five patients and the two well-characterized HPrEpiC primary and LNCaP cancer cell types for model validation.

**Results:**

Principal component analysis presented highest covariability for the four biomarkers 4′,6-diamidino-2-phenylindole, 5-methylcytosine, 5-hydroxymethylcytosine, and alpha-methylacyl-CoA racemase in the epithelial tissue compartment. The panel also showed best performance in discriminating between normal and cancer-like cells in prostate tissues with a sensitivity and specificity of 85%, correctly classified 87% of HPrEpiC as healthy and 99% of LNCaP cells as cancer-like, identified a majority of aberrant cells within histopathologically benign tissues at baseline diagnosis of patients that were later diagnosed with adenocarcinoma. Using k-nearest neighbor classifier with cells from an initial patient biopsy, the biomarkers were able to predict cancer stage and grade of prostatic tissue that occurred at later prostatectomy with 79% accuracy.

**Conclusion:**

Our approach showed favorable diagnostic values to identify the portion and pathological category of aberrant cells in a small subset of sampled tissue cells, correlating with the degree of malignancy beyond baseline.

## INTRODUCTION

PCa is the most common cancer among men and the second leading cause of cancer-related deaths [1]. The standard diagnostic technique for screening of PCa is histopathological review of prostate tissue collected through needle biopsy. 12-core or 14-core transrectal ultrasound-guided prostate needle biopsy (TRUS) is the most prevalent method for initial biopsy from patients with an elevated serum prostate-specific antigen (PSA) level [2]. Currently, although PSA levels and PSA kinetics are gathered, they are not used to define cancer progression on active surveillance [3, 4]. Biopsy classification using a revised version of the original Gleason grading system has emerged as a more meaningful endpoint for monitored men [3–7]. PCa must meet three criteria to be deemed insignificant: the biopsy must receive only Gleason score (GS) 6 (3 + 3) and have a core volume (positive for tumor) of 50% or less (both determined by histology), in combination with an organ confined primary tumor as diagnosed by magnetic resonance imaging (MRI) of the prostate [8]. Higher-grade tumors (GS7 and higher) imply a significantly elevated likelihood of clinical progression [3, 4]. Currently, prostate biopsy remains the only method to ascertain prostate cancer grade. Nevertheless, when low-grade PCa is diagnosed on needle biopsy, there is a risk of undergrading because of a sampling error, which occurs when a higher-grade component in the prostate gland is being missed during the biopsy process [3, 4]. In about 20–30 percent of the time, when a patient has a GS6, there may be higher-grade tissue present in the rest of the prostate. This relatively high frequency of occult tissue has been shown in a meta-analysis of 23 studies (with 100 cases or more) in which ∼35% of all cases were found to have a higher grade at paired radical prostatectomy [9]. Concerns about false-negative results in conjunction with persistent risk factors, such as increased PSA, often leads to repeated biopsies. The negative impact of the limited precision of histopathology may subject cancer-free men to additional invasive biopsy procedures with associated negative effects, including anxiety and the risk of urosepsis [10, 11]. Hence, there is an unmet need for additional biomarkers of prostate malignancy in biopsy tissue to complement current histopathology and PSA to improve diagnostic accuracy and avoid unnecessary rebiopsy. There is a body of evidence, which shows that epigenetic aberrations such as altered DNA methylation and related changes in histone-tail modification patterns amongst other features correlate with several cancer types, including PCa [12, 13]. Epigenetic changes are by now known to occur early in cellular transformation and cancer development. Cancer cells usually display hypermethylation of a relatively small number of single gene promoters, mostly in gene-rich genomic regions termed CpG islands, leading to silencing of certain tumor suppressors involved in cell-cycle regulation, DNA mismatch repair, cellular differentiation, and apoptosis. Hypermethylation of the glutathione S-transferase pi (GSTP1) gene promoter has been observed in nearly 90% of all prostatic carcinomas but not in benign hyperplastic tissue [14, 15]. Other well-characterized hypermethylated genes in PCa *include RASSF1A*, *CDH1* and *CDKN2A*. Promoter hypermethylation is often coexistent with hypomethylation at the global DNA (gDNA) level [16]. A malignant cell can contain 20–60% less genomic 5-methylcytosine (5mC) than its normal counterpart. The loss of methyl groups is achieved mainly by hypomethylation of repetitive DNA sequences, which count for more than 90% of the human genome, including transposable elements (∼48% of genome) such as short and long interspersed nuclear elements (SINES and LINES, respectively), largely acquired as retroviruses throughout evolution [17, 18]. Global demethylation is clinically relevant to progression in a number of cancer types, since loss of global methylation tends to become more pronounced as precancerous lesions advance [19–22]. As for PCa, global hypomethylation correlates with both tumor development and progression [23–26]. This epigenetic phenomenon is often accompanied with the under-presentation of repressive heterochromatin-associated histone marks, predominantly histone-3 lysine-methylation such as H3K27me3 (facultative heterochromatin) and H3K9me3 (constitutive heterochromatin) [13]. Compared to promoter CpGs global hypomethylation has been less investigated as a biomarker, despite the fact that it is ubiquitous and much more pronounced in cancers than is gene-promoter hypermethylation [27, 28]. The global changes in DNA methylation and histone modification patterns can be visualized and quantified by high-resolution light microscopy in conjunction with computer-assisted image analysis [29–33]. Besides DNA methylation also genome-wide changes in cellular DNA hydroxymethylation are associated with various cancers [34, 35]. Recently, we had only briefly disclosed the capability of newly developed 3D high-content analysis to perform DNA methylation phenotyping of cells towards characterization of human prostate tissue heterogeneity [36]. However, biological differences across epigenetic and non-epigenetic biomarkers support the use of several markers in a cancer detection assay. Hence in here we report on using previously developed parallel cell-by-cell analytical techniques —surrounding 3D high-content imaging, chromatin-associated epigenetic in combination with validated PCa-relevant markers, and mathematical-statistical principles for analysis of large imaging data— to generate novel detailed maps of biomarker heterogeneity in patient tissues and assess their potential in PCa diagnosis [30, 37, 38]. The aims of this study were: 1) on cell-by-cell basis to determine the best of two sets of biomarkers that described the variation of variables as parsimoniously as possible using a set of derived uncorrelated variables; 2) assess the capability of the biomarkers chosen in step one to segregate between what we deemed normal and aberrant (malignant cells) based on the tissue of origin; 3) establish the ability of the chosen biomarkers to accurately define the pathological category and follow the progression of PCa in a set of validation data.

## RESULTS

The longitudinal analysis with most of the archived patient specimens comprised tissue samples that were collected at different diagnostic time points, including diagnosis —initial trans-rectal needle biopsy (biopsy 1) and if available a follow-up biopsy (biopsy 2)— and prostatectomy, as shown in Table 1.

**Table 1:** Patient specimens used in the study (including extraction dates)

**Development Specimens**			
**Name**	**Biopsy 1**	**Biopsy 2**	**Prostatectomy**
Patient 1	Atypical glands + small focus of cancer, GS6 (3+3); 03/2011	AC, GS6 (3+3);06/2012	Stage III (pT3a), GS7 (3+4); 12/2012
Patient 2	Lots of AC, GS7 (4+3);11/2012		Stage II (pT2c), GS7 (3+4); 02/2013
Patient 3	Benign; 08/2009	AC, GS7 (4+3); 09/2012	Stage III (pT3a), GS7 (3+4); 12/2012
Patient 4	Lots of AC, GS6 (3+3);02/2012		Stage II (pT2c), GS7 (3+4); 03/2012
Patient 5	Lots of AC, GS6 (3+3);11/2012		Stage III (pT3b), GS6 (3+3); 01/2013
**Validation Specimens**			
Patient 5			Benign tissue distal from tumor region; 01/2013
Patient 6	Lots of AC, GS6 (3+3);5/2014		Stage II (pT2c), GS6 (3+3); 11/2014
Patient 7	Lots of AC, GS6 (3+3);1/2013		Stage II (pT2c), GS6 (3+3); 5/2013
Patient 8	Lots of AC, GS7 (3+4);2/2013		Stage II (pt2c), GS7 (3+4); 4/2013
Patient Z			Benign tissue distal from tumor region; extraction date N/A
HPrEpiC	Isolated from normal human prostate tissue, cytokeratin 18 and 19 positive		
LNCaP	Isolated from human needle biopsy, androgen-sensitive prostate adenocarcinoma cells		

### Differential imaging and analysis of tissues

**Figure 1:**
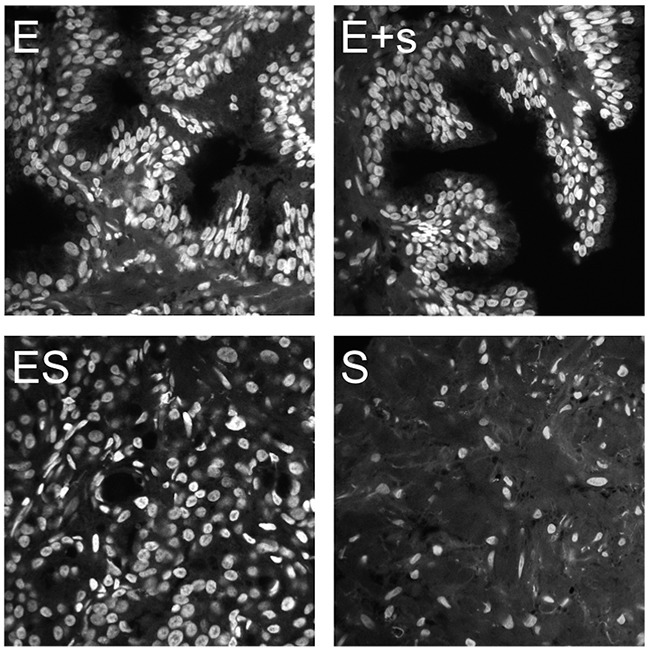
Prostate tissue imaging showing optical mid-sections in the DAPI-channel Imaging was performed to acquire four categories of tissue frames corresponding to a sampling spectrum of epithelial and stromal compartments: epithelia only (E), epithelia with minor bordering stroma (E+s), mixed epithelia and stroma at various ratios (ES), and stroma only (S).

The high-content assay and analysis was performed on the three-dimensional quantitative DNA methylation imaging (3D-qDMI) platform that we had previously introduced [29, 31, 39]. The technology constitutes a multiplexed image-cytometric approach, by which fluorescence signals of simultaneously visualized nuclear targets —generated through established immunocytochemistry and light microscopy— are extracted from 3-D images to visualize and measure changes in global epigenetic markers such as DNA methylation/hydroxymethylation and other molecular targets in thousands of cells in parallel. This capability to analyze cell populations on a per-cell basis is a powerful means in addressing cell population heterogeneity in tissues. Within this context, the application of high-resolution confocal microscopy in the range of 100–500nm allowed us to separately acquire images from the two major tissue compartments, i.e. the epithelium and the stroma, as well as mixed areas that also entailed border regions between the two compartments. Hence for each subject each tissue sample was divided into four categories based on the represented tissue compartments: epithelium only (E), major epithelium with minor stroma (E+s), equal portions of epithelium and stroma (ES), and stroma only (S), as shown in Figure 1.

**Figure 2:**
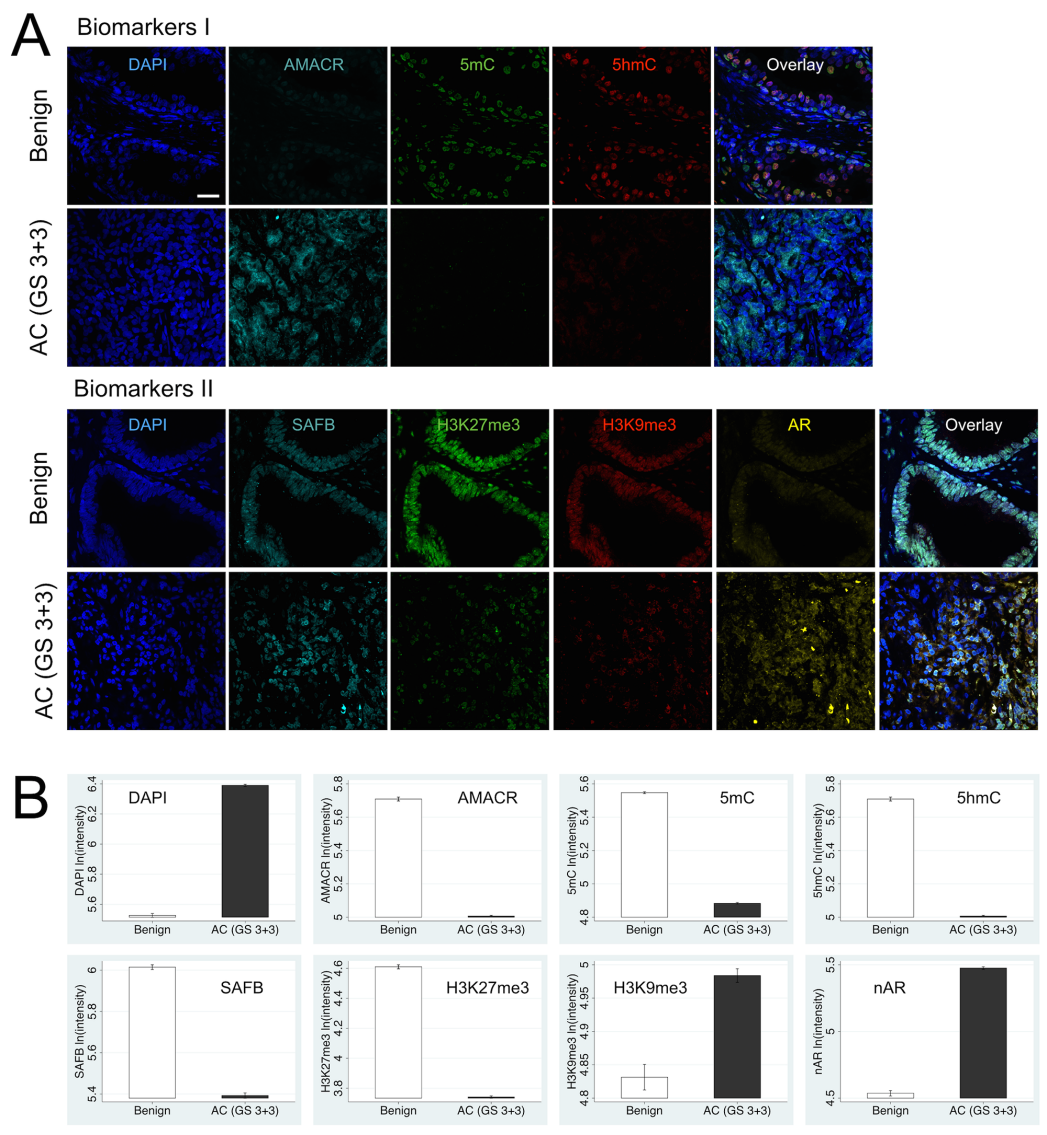
Differential levels and cellular heterogeneity of Biomarkers I and II between biopsied benign and cancerous prostatic tissues (represented by AC with GS6), as visualized by confocal scanning microscopy **(A).** Each marker (false-colored) was recorded in a separate channel. For each tissue sample all channels —including the multi-color overlay image— are presented as maximum intensity projections. **(B)** Quantitative presentation of biomarker levels as bar plots indicate for Biomarkers I: significant increase in DNA content (DAPI) and AMACR levels and simultaneous loss of the two epigenetic DNA modifications 5mC and 5hmC in both basal and luminal epithelial cells in AC versus benign tissue; luminal cells seemingly exhibit a stronger loss of 5hmC compared to basal cells. Biomarkers II: concurrent loss of suppressive chromatin-state marker H3K27me3 and decrease of chromatin-associated SAFB levels, while H3K9me3 and nuclear AR levels are highly elevated in AC versus benign tissue. Scale bar is 10 μm.

Hematoxylin & eosin (H&E) staining of the matching middle sections of the interrogated tissues were used to identify tissue regions for confocal imaging (Supplementary Figure 1). In here we assessed the abundance of two sets of PCa-related biomarkers that are novel in their combination. The first set (Biomarkers I) comprised 4′,6-diamidino-2-phenylindole (DAPI) representing gDNA, the two cytosine variants 5mC and 5-hydroxymethylcytosine (5hmC), and alpha-methylacyl-CoA racemase (AMACR) [40, 41]. The second set (Biomarkers II) included DAPI, the scaffold attachment factor B (SAFB), tri-methylated lysine 9 on histone 3 (H3K9me3), tri-methylated lysine 27 on histone 3 (H3K27me3), and the fraction of the androgen receptor (AR) that was present in the cell nucleus as nAR [42–44]. DAPI was used in both sets also for technical reasons, i.e. to delineate the nuclear region of interest; a standard procedure to enable the segmentation of cell nuclei and the creation of an image mask in order to also retrieve nuclear signals of the other biomarkers in the respective channels, as can be inferred from the methods section. Overall, in adenocarcinoma (AC) versus benign tissue we explored an increase in gDNA content represented by DAPI, as well as AMACR, H3K9me3 and nAR in epithelial cells (Figure 2). Concurrently we could measure a significant decrease in global 5mC, 5hmC, and the chromatin-associated SAFB alongside the suppressive H3K27me3 mark. Interestingly, luminal cells seemingly exhibit a stronger loss of 5hmC compared to basal cells. In reality, cells in benign and cancer tissues display a differential heterogeneity regarding the levels of these biomarkers. For example subsets of cells appear to show more or less SAFB intensity than the average abundance (intensity) of this protein in the epithelial cells of histopathologically (structurally) benign prostate tissues. In comparison, our experience was that tumor areas displayed a higher heterogeneity of SAFB than the morphologically intact (benign) epithelium. Therefore, our imaging results were in agreement with previous findings that had reported similar trends of the individual markers in association with the degree of tissue malignancy [24, 25, 34, 39, 45–48].

### Phenotypic biomarker heterogeneity in tissues

**Figure 3:**
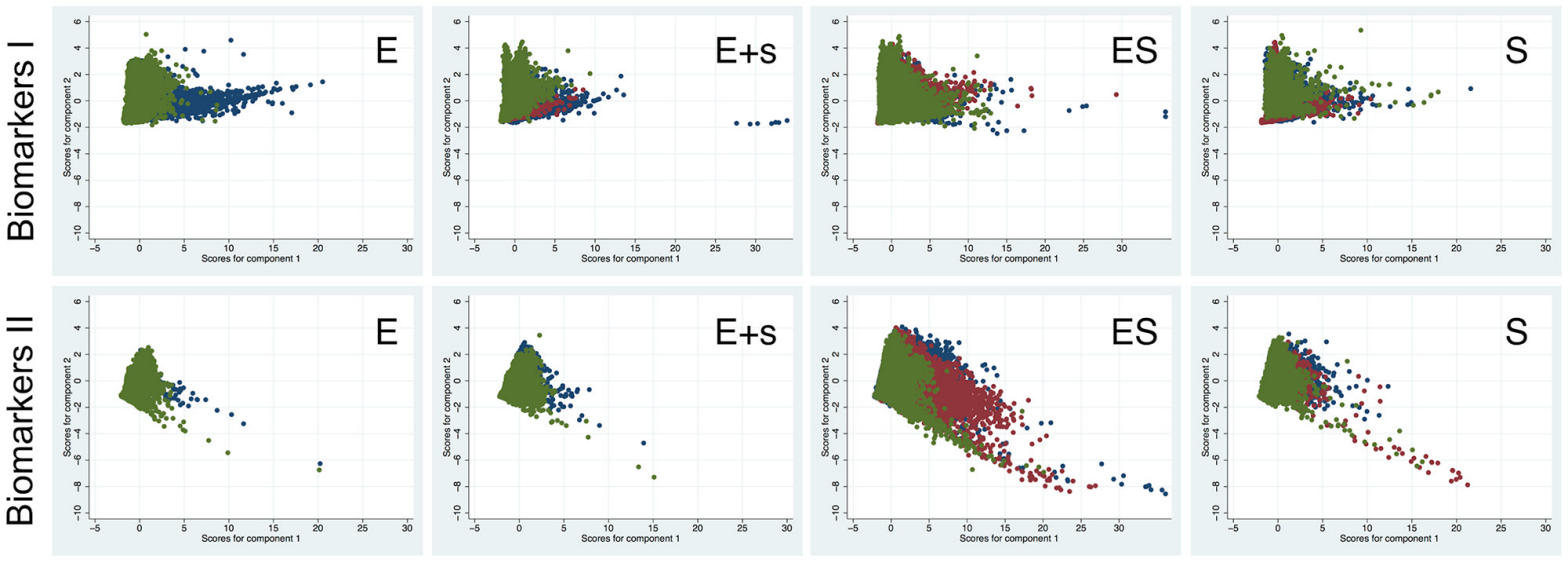
Comparative tissue compartment-specific results of principle component analysis (with two components) for Biomarkers I and II The data was separated into subsets representing the first biopsy (blue), the second biopsy (red), and finally prostatectomy (green). Each dot represents one cell. The results show a high overlap when epithelial and stromal compartments are analyzed together (ES). The overlap is reduced in the case of only a minor involvement of stroma (E+s). The best segregation is seen when the epithelial compartment is analyzed by itself, indicating the highest change (variance) for the analyzed markers. The latter subdata is missing data from two biopsies, as most patients for which epithelial (E) compartments could be analyzed were initially diagnosed with lots of AC and thus only underwent one biopsy prior to prostatectomy.

In addition to our observations at the microscopic level and the report on average changes of biomarker intensities, we were interested in utilizing the asset of cell-by-cell data (gathered in this study) to conduct a systematic quantitative analysis of marker heterogeneity in the different pathological tissues. Because the data were derived from tissues that had been excised at different time-points it was not possible to conduct a continuous assessment of marker-covariance over time. From our previous studies to characterize cell phenotypes in complex cell populations such as differentiating embryonic stem cells (ESC) using big imaging data, we had experienced that principle component analysis is a valuable statistical approach to determine marker variance in multi-parametric assays [38]. Principle component analysis enables the reduction of observed variables, while preserving a large portion of the variance in the data. Using principle component analysis, the newly produced variables were derived in decreasing order of importance pertaining to the amount of variation they explain of the original variables. For example, principle component 1 explains for as much of the variability of the original data as possible. The second principle component explains as much of the remaining variance as possible under assertion that it is not correlated to principle component 1, etc. This reduction is useful as our data could be graphically summarized, instead of exploring the covariance/correlation of the pairwise relationship between the markers within each of the two marker sets (please see Methods section for more detailed explanation). Figure 3 illustrates the degree of compartment-specific data segregation for Biomarkers I (DAPI, AMACR, 5mC, and 5hmC) and Biomarkers II (DAPI, nAR, SAFB, H3K9me3, and H3K27me3) across all exploratory tissue samples from Patients 1 to 5. For Biomarkers I, the highest covariation was found in the epithelial compartment. This variation correlatively diminishes with increasing proportions of stromal regions. As for the abovementioned biomarkers the stromal compartment by itself did not show any significant covariation. The loading matrix (Supplementary Table 1) indicates that the major drivers of principle component 1 (highest variable markers) are 5hmC and AMACR at about equal weight, followed by 5mC, while principle component 2 is dominated by DAPI variables. In comparison, Biomarkers II did not segregate well (Figure 3). In this case principle component 1 was majorly influenced by H3K27me3 and nAR at about equivalent power, followed by SAFB. Principle component 2 was driven by DAPI, as shown in the Supplementary Table 2.

**Figure 4:**
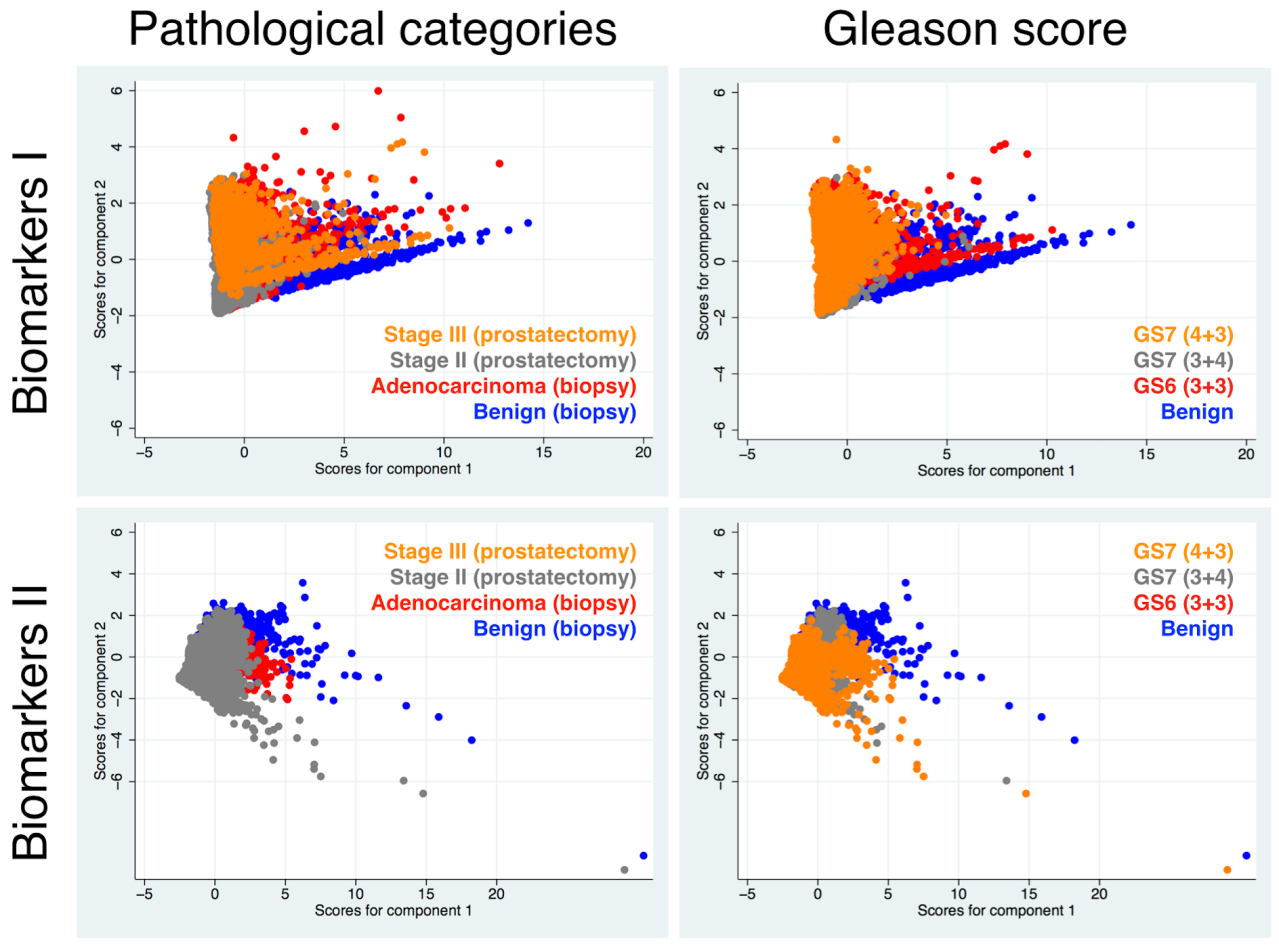
Comparative results of principle component analysis (with two components) for Biomarkers I and II according to clinical diagnoses (pathological categories or GS) The analysis was performed with cells located only in the E-compartment, which showed highest differential results in the first analysis (Figure 3).

Next, we performed principle component analysis using only data derived from the epithelial compartment to explore any covariation of the abovementioned markers in association with tissue malignancy. Tissue malignancy was distinguished either by tissue pathological categories including PCa stages (benign, AC at biopsy, and stage II and stage III cancer at prostatectomy) or by Gleason scores (GS) including GS6 (3+3), GS7 (3+4), and GS7 (4+3). In the relevant Figure 4, the composite plots illustrate that for both biomarker sets there seems to be no significant difference in data segregation between the two tissue classifications. However, there is a strikingly better segregation of the data for Biomarkers I compared with Biomarkers II.

**Figure 5:**
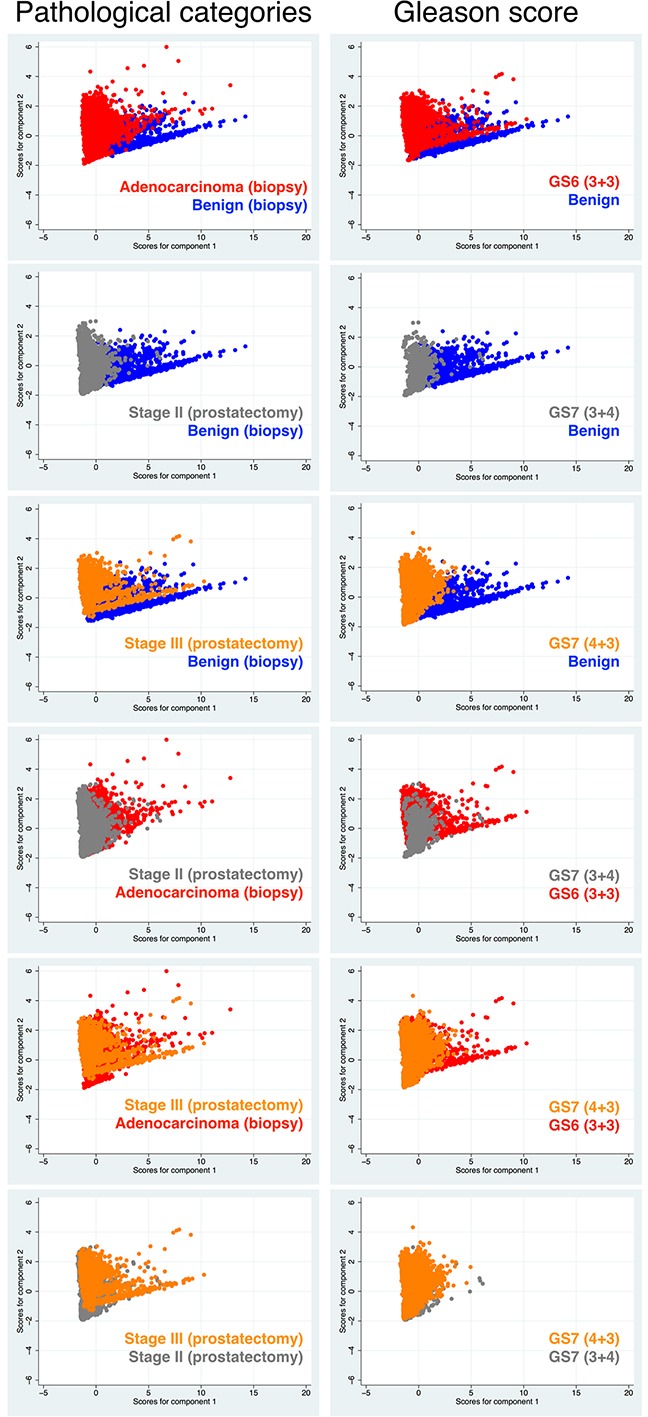
Pairwise comparative results of principle component analysis for Biomarkers I between the two types of disease characteristics (used in diagnosis); i.e. pathological categories including cancer stages versus GS

The pairwise display of the abovementioned results for Biomarkers I revealed more detailed information on the two comparative tissue classifications (Figure 5). The results show significant changes of the markers between the morphologically benign tissue and AC, diagnosed through needle biopsy as well as benign versus stage II and stage III cancers, the latter two conditions diagnosed after prostatectomy. The data for AC is slightly more overlapping with stage II and even more so with stage III, indicating that biopsied AC tissue used in this study must have had very similar values for Biomarkers I compared with prostatectomy samples of patients diagnosed with stage II and even more so with stage III cancer. This is conceivable as stage II plotted against stage III exhibited also a significant overlap. When GS was utilized for tissue characterization, we observed an overall better performance (segregation) of Biomarkers I. Similarly, to when pathological conditions were used as the diagnostic index, benign tissue clearly distinguished itself from cancerous prostate tissues (GS6 and GS7). A significant segregation was also found between GS6 and two GS7 tissue types. However, a notable overlap could be observed between GS (3+4) and the supposedly more aggressive GS (4+3), indicating that only minor differences in Biomarkers I seem to exist between the two grades. Also of note is the fact that of the three benign-labeled tissue samples only one (Patient 3, biopsy 1) showed a clear pattern of segregation from malignant tissue, regardless whether we used pathological conditions or GS to classify the samples. Therefore, this was the only tissue we were confident that it should remain labeled as benign.

### Distinction between cancer and non-cancer cells

**Figure 6:**
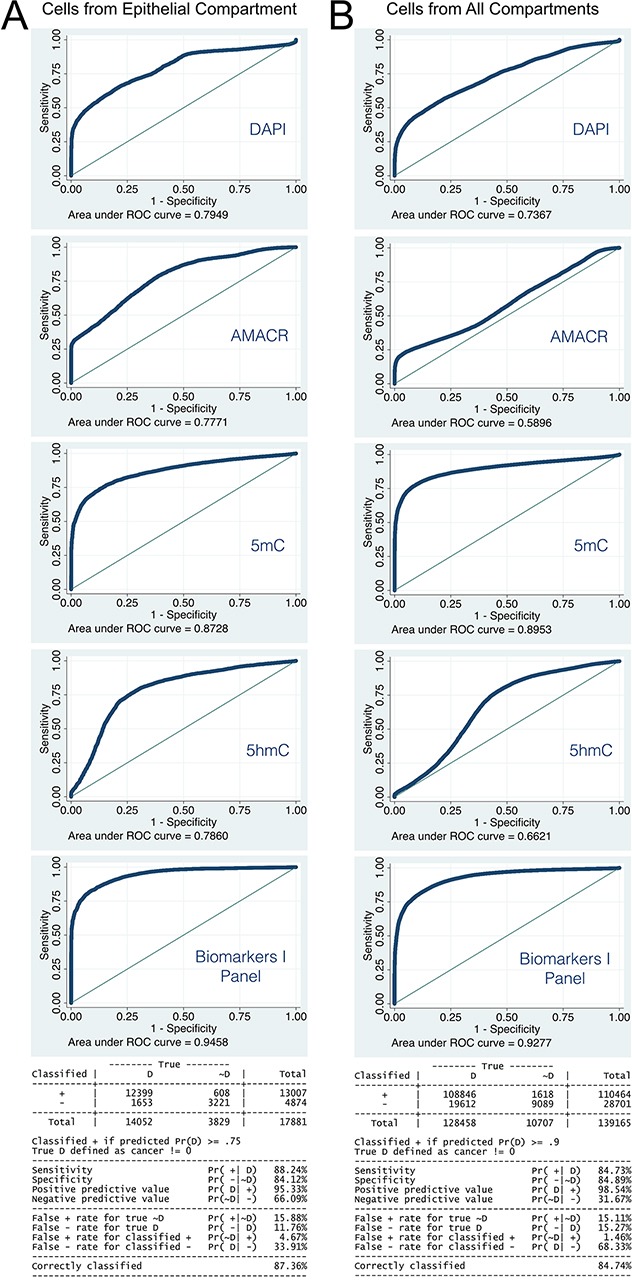
Performance of Logistic regression model with the development data set and Biomarkers I, utilizing epithelial cell only **(A)** and all subsets of all imaged cells **(B).** 5mC shows best performance as single marker in both cases, and is only exceeded by the combined marker panel.

Towards our second objective, we explored two logistic regression models. For both models the data was divided into a developmental subset using tissue data of Patients 1 to 5, and a validation (test) set that comprised data from: a) the two cultured cell types, and Patients 6, 7 and 8, as well as two prostatectomy tissue samples isolated from areas distant from the tumor that had normal appearance based on H&E staining (per expert pathological diagnosis). The latter specimens were from Patient 5 and separately from another patient (Patient Z). For the developmental set, tissue from Patient 3 biopsy 1 —pathologically classified as benign— was used as healthy control. The first logistic regression model was based only on cells that were found within the epithelial tissue compartments. Most of the channel intensities showed to have a significantly skewed distribution. Thus, all channel intensities were log-transformed prior to application in the logistic regression model. In total, we employed a set of 17,881 cells of which 3,829 were rated as normal and 14,052 as aberrant (cancerous) cells. Using univariate logistic regression, we established that individual markers from the Biomarkers I panel were all significantly associated with aberrant cells (Supplementary Table 1). However, upon assessment of goodness-of-fit analysis, only 5mC showed excellent discrimination power (epithelial tissue only: area under a curve of the Receiver Operating Characteristics (AUC ROC)=0.87; both tissue compartments together: AUC ROC=0.89; Figure 6). Once we established that essentially all biomarkers had a significant association with cancer we conducted multivariate logistic regression using all four members of the Biomarkers I panel (Table 2). Log-transformed unit changes in marker intensities resulted in enormous alterations in the odds ratio (OR) for cancer: for DAPI a 7-fold increase (95% Cred. Int.: 6.7–7.8 OR), for AMACR an 80% decrease (95% Cred. Int: 82%–77%), for 5mC a 98% decrease (95% Cred. Int: 97.5%–99.2%), and for 5hmC a 3-fold decrease (95% Cred. Int: 2.5–3.4 OR). Based on the probability of cancer using a cutoff point of 0.75, this model resulted in 88% sensitivity and 84% specificity, whereby 87% of the cells were accurately classified (Figure 6A). Overall across various probability cutoffs we observed an outstanding discrimination between normal and abnormal cells of AUC ROC=0.95 with this logistic model. For further explanation please refer to Methods section *(Statistical learning methods and diagnosis, staging and prognosis of cancer)*.

**Table 2:** Logistic regression model coefficients for epithelial cells only

Marker	Odds Ratio	Std. Err.	z	P>z	[95% Conf.	Interval]
lnDAPI	7.224606	0.2921743	48.9	<0.0001	6.674063	7.820565
lnAMACR	0.199478	0.0132372	−24.29	<0.0001	0.1751499	0.2271852
ln5mC	0.0199724	0.0017131	−45.63	<0.0001	0.0168818	0.0236286
ln5hmC	2.95159	0.2216339	14.41	<0.0001	2.547649	3.419578
_cons	490131.5	201505.3	31.87	<0.0001	218958.8	1097142

The validation subset of data showed that our logistic model classified 87% of the HPrEpiC as non-cancerous (normal). Analogously, 99% of the LNCaP cells were classified as cancerous (Table 3A). Interestingly, the supposedly “normal/benign” tissues that were isolated distally from the tumor region in Patients 5 and Z during prostatectomy, were classified as containing an overwhelming portion of cells that exhibited an aberrant (cancer-type) Biomarker I profile: 99.9% in Patient 5 and 77% in Patient Z (Table 3B). In data of Patient 6, which was not part of our development model, we identified 87% of cells in the first biopsy (B1) sample and 93% of the cells in the prostatectomy (P) sample as being transformed (cancerous, Table 3C). Finally, for Patient 7 the logistic model estimated that 99% of cells isolated during initial biopsy were cancer-like. The model based on purely cells from the epithelial compartment estimated that 94% of the cells from Patient 8 were abnormal (Table 3D).

**Table 3:** Predictions of the logistic model based on epithelial cells only

A	Presence of cancer cell		
ID	No	Yes	Total
HPrEpiC	1,800	266	2,066
%	87.12	12.88	100.00
LNCaP	148	17,289	17,437
%	0.85	99.15	100.00
**B**	**Presence of cancer cell in patients 5 and Z**		
**ID**	**No**	**Yes**	**Total**
Patient 5	3	11,582	11,585
%	0.03	99.97	100
Patient Z	2,168	7,206	9,374
%	23.13	76.87	100
**C**	**Presence of cancer cell in patient 6**		
**Phase**	**No**	**Yes**	**Total**
B1	359	2,360	2,719
%	13.2	86.8	100
P	1,488	18,634	20,122
%	7.39	92.61	100
**D**	**Presence of cancer cell in patients 7 and 8**		
**ID**	**No**	**Yes**	**Total**
Patient 7	71	5,622	5,693
%	1.25	98.75	100
Patient 8	519	7,996	8,515
%	6.1	93.9	100

(A) cancer and benign cell types; (B) pathologically defined benign tissue isolated during prostatectomy of patients 5 and Z; (C) patient 6: prediction of cancer at time point of tissue isolation. (D) tissue isolated during initial biopsy from patients 7 and 8.

The second logistic regression model was developed using the pool of all imaged cells (139,165) from Patients 1 to 5 (development dataset, Table 4). This model had a cutoff point of 0.9, resulting in 85% sensitivity and specificity (Figure 6): 85% of cells were accurately classified (Figure 6A). Again, the overall discrimination power of the model was outstanding with AUC ROC=0.92–0.94. The model identified 81% of the HPrEpiC as normal and in contrast 99% of the LNCaP cells as cancer-like (transformed, Table 5A). The two “supposedly benign” tissues were indicated to contain 100% (Patient 5) and 84% (Patient Z) aberrant cells (Table 5B). For Patients 6, 7, and 8, the model classified 92%/98%/92% of cells respectively as aberrant at baseline. For Patient 6 also 95% of cells in the prostatectomy were classified as aberrant (Table 5C and 5D).

**Table 4:** Logistic regression model coefficients for all cells

Marker	Odds Ratio	Std. Err.	z	P>z	[95% Conf.	Interval]
lnDAPI	6.459606	0.1339882	89.94	<0.0001	6.20226	6.727629
lnAMACR	0.4555956	0.0120028	−29.84	<0.0001	0.4326676	0.4797386
ln5mC	0.0290629	0.0009604	−107.08	<0.0001	0.0272403	0.0310075
ln5hmC	3.055547	0.0929042	36.74	<0.0001	2.878778	3.243171
_cons	3845.418	650.8334	48.77	<0.0001	2759.806	5358.073

**Table 5:** Predictions of logistic model based on all imaged cells

A	Presence of cancer cell		
ID	No	Yes	Total
HPrEpiC	1,674	392	2,066
%	81.03	18.97	100
LNCaP	159	17,278	17,437
%	0.91	99.09	100
**B**	**Presence of cancer**		
**ID**	**No**	**Yes**	**Total**
Patient 5	6	11,579	11,585
%	0.05	99.95	100
Patient Z	704	7,811	8,515
%	8.27	91.73	100
**C**	**Presence of cancer cell (Patient 6)**		
**Phase**	**No**	**Yes**	**Total**
B1	209	2,510	2,719
%	7.69	92.31	100
P	1,077	19,045	20,122
%	5.35	94.65	100
**D**	**Presence of cancer cell**		
**ID**	**No**	**Yes**	**Total**
Patient 7	96	5,597	5,693
%	1.69	98.31	100
Patient 8	704	7,811	8,515
%	8.27	91.73	100

### Modeling the prediction of indolent versus progressive cancer

**Figure 7:**
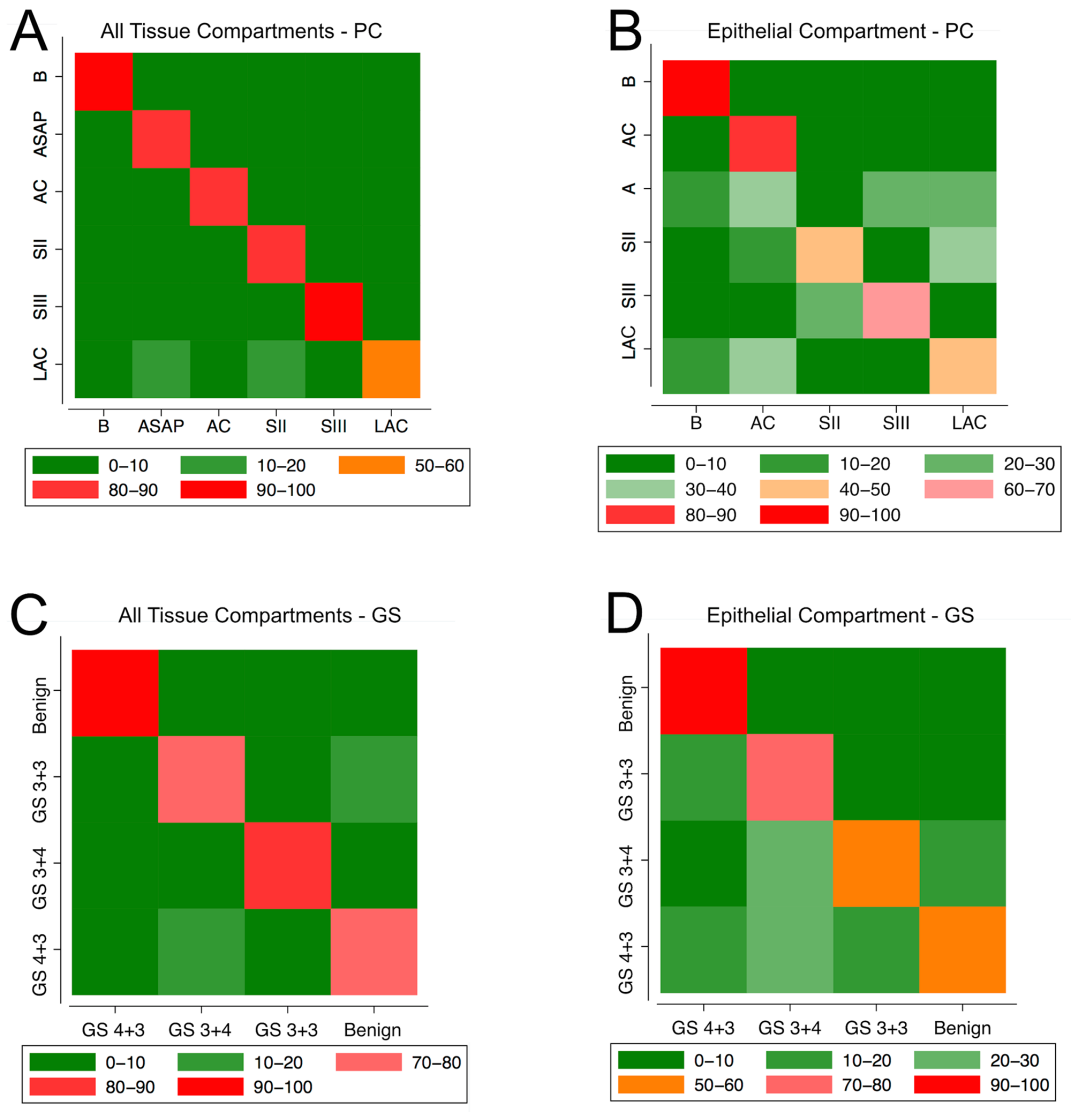
Heat maps representing the performance of the KNN classification

Towards the third goal of our study, our analyses focused on the prediction of a) the pathological categories of the tissue, and b) cancer aggressiveness and disease progression. Hereby the following six pathological categories were used: benign, atypical small acinar proliferation (ASAP), adenocarcinoma (AC), lots of adenocarcinoma (LAC), as diagnosed at biopsy, and stage II and stage III as diagnosed at prostatectomy. Cancer aggressiveness and disease progression were defined by the four GS: benign (B) or no score (Group 1), score 6 (3+3) (Group 2), score 7 (3+4) (Group 3), and score 7 (4+3) (Group 4). For the purpose of developing predictive models we applied *k*-nearest neighbor (KNN) classifier, a machine-learning tool. Both, in development and validation, we employed the same strategy (tissue data) as for the logistic regression model: first only epithelial cells and subsequently subsets of random cells across both tissues compartments were recruited in order to generate two unique models per category scheme. For predictive modeling of tissue pathological categories we drafted a total of 17,881 imaged epithelial cells. Of these, 3,829 cells were recruited from benign (per logistic regression analysis) tissue sources. The KNN classification identified 91% of these cells as benign or non-cancerous. 84% of the 11,712 cells in ASAP were correctly classified (Figure 7). Overall, the KNN was able to faithfully affiliate tissue pathological category with 67% accuracy (Figure 7B).

Once the KNN classification was established we proceeded with model validation. Of the 2,066 HPrEpiC KNN identified 87% as benign (Table 6A). Of the 17,437 LNCaP cells 62% were found to belong to stage II and 22% were classified as LAC-type cells. In the assumingly healthy prostatectomy-derived tissue from Patient 5, who had been diagnosed with stage III (pT3b) PCa, KNN associated the majority of cells (65%) with LAC, closely followed by 20% (on average) of stage III-type cells. For Patient Z the model classified 60% of the cells as LAC-type, followed by 28% rated as stage III-type (Table 6B). For Patient 6 we estimated that from the 2,719 cells obtained during biopsy 39% and 29% were classified as stage III and LAC-type, respectively. Similarly, from the 20,122 cells isolated during prostatectomy, 51% were classified as stage III cancer and 43% as LAC-type (Table 6C). Finally, the majority of cells in Patients 7 (68%) and 8 (49%) were also classified as stage III-like cancer cells and 49% of the cells from Patient 8 were identified as stage III, followed by smaller portions of LAC-type cells: 17% and 22%, respectively (Table 6D).

**Table 6:** Validation of KNN classification for predicting tissue pathological categories (including cancer stages) using epithelial cells only and Biomarkers I

A	Classification					
ID	B	ASAP	Stage II	Stage III	LAC	Total
HPrEpiC	1,798	29	49	102	88	2,066
%	87.03	1.4	2.37	4.94	4.26	100
LNCaP	128	1,070	10,775	1,587	3,877	17,437
%	0.73	6.14	61.79	9.1	22.23	100
**B**	**Classification**					
**ID**	**B**	**ASAP**	**Stage II**	**Stage III**	**LAC**	**Total**
Patient 5	2	61	1,687	2,355	7,480	11,585
%	0.02	0.53	14.56	20.33	64.57	100
Patient Z	207	832	73	2,652	5,610	9,374
%	2.21	8.88	0.78	28.29	59.85	100
**C**	**Classification**					
**Phase**	**B**	**ASAP**	**Stage II**	**Stage III**	**LAC**	**Total**
B1	589	202	87	1,052	789	2,719
%	21.66	7.43	3.2	38.69	29.02	100
P	437	650	74	10,270	8,691	20,122
%	2.17	3.23	0.37	51.04	43.19	100
**D**	**Classification**					
**ID**	**B**	**ASAP**	**Stage II**	**Stage III**	**LAC**	**Total**
Patient 7	9	250	536	3,851	983	5,629
%	0.16	4.44	9.52	68.41	17.46	100
Patient 8	232	1,038	1,215	4,136	1,894	8,515
%	2.72	12.19	14.27	48.57	22.24	100

To simplify the use of our KNN classification we decided to use all the cells at our disposal without discrimination based on tissue (compartment) origin. One limitation of the KNN methodology is that it requires immense meta-data storage capabilities [50]. Hence in order to make our development dataset more portable we determined that 30,000 cells from both tissue compartments (epithelium and stroma) and across all six pathological categories was the optimal number of cells that needed to be randomly chosen for obtaining the most accurate KNN classification. Even though we used either solely epithelial cells or a subset of all imaged cells (139,165 cells) for the development of the KNN classifications, we assessed model accuracy with all 139,165 cells from our development dataset (Figure 7A). KNN failed to classify a marginal portion of 4% (∼5.500) cells. From the remaining 96% of cells, KNN classification faithfully affiliated 79% of all cells. In detail, the analysis of our validation dataset showed that 80% of the HPrEpiC were correctly identified as being normal (benign), and 87% of the LNCaP cells were classified as stage II cancer cells (Table 7A). The assumingly benign prostatectomy tissues isolated from PCa patients were indicated as follows: Patient 5 tissue was diagnosed of harboring 68% stage II cells followed by 24% LAC-type cells (Table 7B). Subject Z tissue was found to consist of 44% stage II cells and an almost equal portion (38%) of LAC cells. Further, for Patient 6, biopsy 1 was populated with a majority (45%) of LAC-type cells and an additional 19% of stage II cells. The prostatectomy tissue seemed to host a majority (47%) of LAC cells and a smaller fraction (37%) of stage II cells (Table 7C). Cells from Patient 7′s first biopsy —initially diagnosed with stage II (pT2c) adenocarcinoma— were classified to be 63% of LAC-type and 28% stage II-type, with a minor stage III component (7%). The patient had not progressed beyond pT2c at prostatectomy (Table 1). In comparison, almost 38% and 29% of the cells from Patient 8′s biopsy —initially diagnosed with stage II (pT2c) adenocarcinoma— were identified to be of LAC and stage II-type, respectively, with a larger stage III fraction (22%) (Table 7D). Also this patient had not progressed further at prostatectomy (Table 1).

**Table 7:** KNN classification-based predictions of pathological categories with subsets of 30,000 cells and Biomarkers I

A	Classification						
ID	B	ASAP	AC	Stage II	Stage III	LAC	Total
HPrEpiC	1,613	20	221	35	16	106	2,011
%	80.21	0.99	10.99	1.74	0.8	5.27	100
LNCaP	78	262	430	14,681	264	1,220	16,935
%	0.46	1.55	2.54	86.69	1.56	7.2	100
**B**	**Classification**						
**ID**	**B**	**ASAP**	**AC**	**Stage II**	**Stage III**	**LAC**	**Total**
Patient 5	0	1	24	7,693	878	2,716	11,312
%	0	0.01	0.21	68.01	7.76	24.01	100
Patient Z	201	1,062	35	3,935	265	3,415	8,913
%	2.26	11.92	0.39	44.15	2.97	38.31	100
**C**	**Classification**						
**Phase**	**B**	**ASAP**	**AC**	**Stage II**	**Stage III**	**LAC**	**Total**
B1	277	131	416	453	56	1,102	2,435
%	11.38	5.38	17.08	18.6	2.3	45.26	100
P	330	443	263	6,164	1,705	7,874	16,779
%	1.97	2.64	1.57	36.74	10.16	46.93	100
**D**	**Classification**						
**ID**	**B**	**ASAP**	**AC**	**Stage II**	**Stage III**	**LAC**	**Total**
Patient 7	7	11	32	1,568	411	3,495	5,524
%	0.13	0.2	0.58	28.39	7.44	63.27	100
Patient 8	142	140	578	2,291	1,779	2,968	7,898
%	1.8	1.77	7.32	29.01	22.52	37.58	100

The predictive power of the KNN algorithm considering GS was estimated using the same two subgroup data as for tissue pathological categories: a) purely epithelial cells and b) subsets of 30,000 epithelial and stromal cells. For both approaches cells across all samples of Patients 1 to 5 were used (Figure 7). The KNN based exclusively on epithelial cells correctly classified 67% of cells in the development dataset (Figure 7B). During validation we found that 88% of HPrEpiC were classified as benign/normal (Group 1), and the absolute majority of the LNCaP cells were identified as cancer cells: 67% were associated with the more advanced GS 7 (3+4) (Group 3) and 23% even with GS 7 (4+3) (Group 4), as shown in Table 8A. Again, KNN classified the supposedly healthy prostatectomy tissue from Patients 5 and Z to be populated with an absolute majority (72% and 68%, respectively) of the more aggressive Group 4 cells (Table 8B). For Patient 6 —diagnosed with adenocarcinoma GS (3+3)— biopsied tissue was almost equally partitioned into 32% Group 3 cells and 29% Group 4 cells, followed by smaller portions of the Group 1 (23%) and Group 2 cells (16%) (Table 8C). This trend seemed to be even more advanced at the time of prostatectomy (patient was diagnosed with GS (3+3)), where 41% of the 20,122 analyzed cells were classified as Group 3 and a larger portion (54%) as the more aggressive Group 4 cells. As for patient 7 —initially diagnosed with adenocarcinoma GS (3+3)— the cells were almost equally assigned to GS6 (40%) and GS (3+4) (44%) with a minor portion of GS (4+3) cells (15%) (Table 8D). At the time of prostatectomy the patient had not significantly progressed and had been diagnosed with GS (3+3) in 40% of total tissue, however with perineural invasion present (Table 1). In the case of Patient 8 KNN detailed the biopsied tissue to contain a majority (54%) of GS (3+4) cells closely followed by a large fraction (38%) of GS (3+3) cells. The patient was diagnosed with GS7 (3+4) at initial biopsy and later at prostatectomy.

**Table 8:** Validation of KNN classification for predicting GS based on epithelial cells only with Biomarkers I

A	Classification				
ID	B	3+3	3+4	4+3	Total
HPrEpiC	1,820	27	140	79	2,066
%	88.09	1.31	6.78	3.82	100
LNCaP	154	1,535	11,697	4,051	17,437
%	0.88	8.8	67.08	23.23	100
**B**	**Classification**				
**ID**	**B**	**3+3**	**3+4**	**4+3**	**Total**
Patient 5	4	49	3,234	8,298	11,585
%	0.03	0.42	27.92	71.63	100
Patient Z	210	679	2,113	6,372	9,374
%	2.24	7.24	22.54	67.98	100
**C**	**Classification**				
**Phase**	**B**	**3+3**	**3+4**	**4+3**	**Total**
B1	638	441	865	775	2,719
%	23.46	16.22	31.81	28.5	100
P	420	554	8,239	10,909	20,122
%	2.09	2.75	40.95	54.21	100
**D**	**Classification**				
**ID**	**B**	**3+3**	**3+4**	**4+3**	**Total**
Patient 7	8	2,226	2,516	879	5,629
%	0.14	39.55	44.7	15.62	100
Patient 8	239	3,278	4,584	414	8,515
%	2.81	38.5	53.83	4.86	100

The performance of the KNN classification using randomly selected subsets of 30,000 cells was on average 79% accurate within the development set of 139,165 cells (Figure 7C). 81% of the HPrEpiC were found to be non-cancerous (Group 1), and conversely 77% of the LNCaP cells were assigned to Group 3 (Table 9A). The supposedly benign prostatectomy tissue of Patient 5 harbored 55% Group 3 cells and 45% Group 4 cells. For Patient Z the associations were reciprocal: 52% Group 4 and 37% to Group 3 cells (Table 9B). Lastly for Patient 6, biopsy 1 showed a larger number (59%) of Group 4 cells. This portion increased to 69% at prostatectomy (Table 9C). The majority of cells from Patient 7 (67%) and Patient 8 (48%) were found to be of Group 4-type (GS (4+3)) closely followed by Group 3 cells (Patient 8: 30%; Patient 9: 36%; Table 9D).

**Table 9:** KNN classification-based predictions of GS with subsets of 30,000 cells and Biomarkers I

A	Classification				
ID	B	3+3	3+4	4+3	Total
HPrEpiC	1,672	166	33	195	2,066
%	80.93	8.03	1.6	9.44	100
LNCaP	85	507	13,482	3,363	17,437
%	0.49	2.91	77.32	19.29	100
**B**	**Classification**				
**ID**	**B**	**3+3**	**3+4**	**4+3**	**Total**
Patient 5	0	67	6,323	5,195	11,585
%	0	0.58	54.58	44.84	100
Patient Z	222	792	3,500	4,860	9,374
%	2.37	8.45	37.34	51.85	100
**C**	**Classification**				
**Phase**	**B**	**3+3**	**3+4**	**4+3**	**Total**
**B1**	**254**	**329**	**539**	**1,597**	**2,719**
%	9.34	12.1	19.82	58.73	100
%	1.71	2.67	26.58	69.04	100
**D**	**Classification**				
**ID**	**B**	**3+3**	**3+4**	**4+3**	**Total**
Patient 7	13	117	1,705	3,794	5,629
%	0.23	2.08	30.29	67.4	100
Patient 8	183	1,176	3,086	4,070	8,515
%	2.15	13.81	36.24	47.8	100

With Biomarkers II we took the identical approach to the first panel. Briefly, the channel values for all markers had to be log-transformed because the data did not show a normal distribution. We then conducted logistic regression analysis in order to predict the specificity and sensitivity of the second panel for successful prediction of presence or absence of aberrant cells in a subset of all imaged cells. Then the model was applied to the same training dataset. When considering only epithelial cells in the analysis with the second panel, unfortunately were not able to generate data for all the pathological categories applied in here for Biomarkers I. Only benign as well as stage II-type and stage III-type cells were present. Nevertheless, the model was able to achieve a fairly good sensitivity (84%) and specificity (81%) over the quota of all 153,000 imaged cells. However, the model trained with Biomarkers II failed to identify HPrEpiC as benign. Instead it characterized 98% of these primary cells as being malignant. This was also the case when the pool of all imaged cells of Patients 1 to 5 was used for model development. As abovementioned, since for Biomarkers II there were only 3 pathological categories available when using cells from the epithelial compartment for model development, we decided to apply KNN with cells from all tissue compartments. While we obtained almost 100% accuracy in correctly matching the cells with their respective pathological category, the KNN classification developed with Biomarkers II also failed to identify the majority of HPrEpiC as benign. Instead, 64% of HPrEpiC were classified as stage III-type cancer cells while only 22% were faithfully classified as benign. Hence we did not pursue the further assessment of Biomarkers II.

## DISCUSSION

The aims of our study were to assess the potential of two sets of biomarkers in conjunction with 3D high-content imaging and fluorescence readout towards: a) early detection of aberrant cells in prostatic tissue using logistic regression analysis, and b) consequent prediction of cancer progression based on cell composition in biopsied tissue at baseline, powered by KNN classification. The novelty of this study was two-fold: (i) in the combinatorial use of global epigenetic features (DNA methylation/hydroxymethylation and two well-characterized histone-tail modifications) together with established markers of prostate pathology, and (ii) the application of extracted cell-by-cell information for diagnostic/prognostic disease modeling. The notion for our approach arised from the fact that epigenetic changes occur early in cancerogenesis and that such changes have been also correlated with disease progression. Specifically, the loss of DNA methylation/hydroxymethylation and trimethylation at lysine 9 and 27 of histone 3 have been extensively shown to be associated with the different stages of PCa. Also loss of chromatin-associated proteins such as SAFB have been reported in connection with PCa, as well as an increase in AMACR and the nuclear portion of cellular AR [24, 25, 34, 39, 45–48]. As we have shown with Figure 2, measurement of average values for biomarker abundance usually does not reflect cellular heterogeneity within tissues, and can only present average biomarker trends when comparing different pathological cases. Because of this fact we intended to exploit the valuable capability of highly-parallel single-cell analysis provided by high-resolution microscopy [29, 31, 38] in combination with sophisticated statistical tools for more complex data. In the exploratory phase of this study and utilizing principal component analysis to graphically assess the variance of the data, we established that Biomarkers I including 5mC and 5hmC presented a better partitioning of the data (Figure 3), and later on showed a better performance (specificity/sensitivity) in both cases when tumor specifications and GS were used as reference pathological tissue classifications. Therefore, we decided to initially proceed with data analysis of Biomarkers I. Perhaps the biggest complication of this study was the fact that we were lacking a truly normal prostate tissue sample. Nevertheless, through careful consideration of our three samples labeled by expert pathologist as benign, and using PCA analysis we identified one that most closely resembled a pattern of dispersion most indicative of the possibility of normal tissue. Further validation of our approach was the fact that our goodness-fit assessment of both logistic and KNN classification showed outstanding results.

Both logistic regression and KNN classifiers employed in our study had been previously applied for the purpose of early detection of cancer [49]. However, in those studies both methods had been either used to cross-validate the approaches or compared against each other to assess their respective predictive performance [40, 41, 49]. Conceivably the most challenging aspect of prior analyses had been narrowing down the number of predictors since KNN classifier only performs well with a very limited number of independent variables [42]. In our study, the two sets of biomarkers were chosen in part based on scientific rationale and in part based on clinical validation for a majority of cancers and especially for PCa (as referenced above) rather than their statistical performance. The statistical methodology we present here differs from previously reported applications in at least two aspects. First, instead of using the two approaches for cross-validation, logistic regression was used to characterize the composition of cells which would be indicative of the presence or absence of PCa-specific aberrations. Subsequently, KNN classifier was utilized to correlate these aberrations with pathological category/grade of cancer. Thus, the two approaches operated collectively in the prediction of PCa presence and severity. Second and final, in abovementioned previous studies, each observation had been relating to one patient sample, whereas in our case each observation was related to a single cell within a tissue sample. Thus, our approach is a true cell-by-cell approach and the derived model predicts the presence or absence of aberrant cancer-like cells, leading to the characterization of the overall tissue sample based on its composition of classified cells and the resulting portion of aberrant cells. However, for each of the individual cells in our sample we did not have a full and comprehensive status. We were only aware of the status of each of the tissue samples based on the diagnosis performed by a professional pathologist. To bridge the gap between the individual cells and the originating tissue we adopted a fuzzy logic summary of the results where the largest share of cells would represent the probability of that subject being overall affected by malignancy or being free of it [50]. Hence we established a more detailed profile of a patient sample with a probabilistic outcome that may allow for prediction of cancer progression (prognostics) rather than only generating a diagnostic snapshot. Nevertheless, future studies will be required in which we can identify possible probabilistic cutoffs for the proportion of aberrant cells that will enable a more deterministic outcome in regards to the presence or absence of malignancy. From a data-structure perspective, in the first phase of analysis, we used logistic regression, which implied a binary outcome —presence (value 0) or absence (value 1) of malignance— for each cell. In the second and final step of our analysis, we shifted towards a polychotomous outcome by which we either predicted the resulting pathological category or grade of cancer based on the proportions of the different subtypes of cells. As part of the comprehensive statistical analyses and in order to determine whether the chosen marker panels serve as good predictors of prostate cancer stage and grade, we conducted a preliminary assessment of goodness-of-fit analysis [51]. From the estimates of the coefficients of the logistic model (Tables 2 and 4) it became clear that all of the four markers (DAPI, AMACR, 5mC and 5hmC) were significantly associated with the likelihood of cancer. Furthermore, the panel revealed our optimized cutoff probability point on per-cell bases yielded an excellent sensitivity and specificity with majority of the cells being accurately classified. Finally, the estimated AUC ROCs in case of both models yielded a value above 0.9, which according to Hosmer and Lemeshaw, indicates outstanding discrimination capability of the logistic model (Figure 6) [51]. Based on above mentioned metrics, we were confident that our logistic model had excellent power of predicting the outcome of presence or absence of malignancy.

While KNN classifier is not as developed in its measures of goodness-of-fit as logistic regression, it still has some elementary measures of accuracy of the classification. KNN classifiers based on all cells (Figure 7A and 7C) identified most cells with correct tissue pathological categories and Gleason score with accuracy higher than 80% percent. While KNN classifier based on epithelial cells (Figure 7B and 7D) was slightly inferior to the KNN classifier based on all cells, it was still able to classify most cells with above 40% accuracy. Once we established that we have a reasonable good classifier we proceeded with validating the classifiers using a dataset of two cultured cell types and five tissue samples. Our validation strategy for Biomarkers I in conjunction with both classification approaches can be divided into three steps.

### Detection of benign and aberrant cells in benign prostate tissue

In the first step, our goal was to assess whether prostate tissue isolated from a patient diagnosed to have PCa and classified as “normal” by an expert pathologist, would contain aberrant cells. By that we were trying to establish if Biomarkers I would indicate any signatures of malignancy present in a tissue that was labeled as benign according to conventional pathological features. Not only we were able to detect aberrant cells in these “benign” validation samples but we also accurately correlated these aberrant cells with pathological category and GS of the patients’ tumors. The reader should note that while these benign samples were not included in our development datasets, other malignant tissue from the same patients was. This fact underlines the capability of cell-by-cell characterization with Biomarkers I of identifying already existing cancer-like aberrations in occult tissue beyond the tumor region. This feature would be in agreement with the concept of field cancerization, also known as a field effect or field defect, which suggests that detectable epigenetic alterations occur in histopathologically nonmalignant tissue that is contiguous with cancerous tissue [43, 44]. Epigenetic changes in field cancerization not only involve hypermethylation, but also hypomethylation [52]. Because the field, in which altered cells reside can extend beyond the morphologically evident tumor into the tumor environment, current histopathological practices may result in high false-negative diagnoses. However, with the implementation of epigenetic biomarkers there is hope to improve this aspect: biopsy samples taken from outside the cancerous tumor focus that result in negative pathology findings with current practices may produce a positive diagnostic result when epigenetic features are included into tissue analysis and classification. This may also reduce the burden of repeat biopsies. Within this context, our approach with Biomarkers I could potentially raise the chance of detecting malignancy even if neoplastic tissue (as per current definition) is being missed during needle biopsy (currently in 20–30 % of cases). This would lead to decreasing the chance of false-negative calls, thus speaking in favor of a better early PCa diagnosis.

### Validation and prediction of cancer grading using known cell culture models

Unfortunately, for a direct comparison of malignant versus benign tissue, we were unable to obtain a more reliable normal reference, i.e. prostate tissue samples from patients that were not diagnosed as having PCa or any other malignancy in follow-up examinations. Therefore, in the second step we focused on using well-established and comprehensively analyzed cell cultures for comparative validation: LNCaP cells as a positive control and primary HPrEpiC representing normal non-transformed cells of epithelial origin. Again, our classification methods accurately identified HPrEpiC and LNCaP cells to be normal and aberrant, respectively, with highest probabilities (90% and 99%).

### Validation and prediction of cancer

In step three of our validation we tried to assess an equally important matter, i.e. the capability of our single-cell imaging approach with Biomarkers I to predict tissue/cancer progression with data obtained at baseline and projected towards prostatectomy. Also in this regard both classifications were able to correctly predict PCa indolence in all three validation Patients 6, 7, and 8 down to stage and GS, as no change was reported for either specification at later prostatectomy. In summary, both mathematical-statistical classification methods demonstrated excellent predictive capabilities in conjunction with cancer pathological categories (including staging), whereas KNN resting on GS revealed mixed scores that were not presented by pathological (expert) test results. The latter needs to be further investigated, whether these results could reflect or explain the current controversies in the field of Gleason scoring. With the advent of thin core biopsy and radical prostatectomy it has become clear that, as originally defined, some aspects of the GS system do not accord with subsequent clinical behavior. Even though the system has been subject to changes since 2005, areas of controversy remain regarding GS6 and in particular GS7 [53]. Donald Gleason himself noted exact reproducibility of score in 50% of needle biopsies and a variance of ±1 score in 85%, similar to the findings of others [54]. Finally, and critical in terms of practicality, we experienced that the performance of Biomarkers I-based models using randomized groups of cells across both compartments (epithelium and stroma) for cell-by-cell classification of tissues are independent from the location of the analyzed cells. Thus our approach resulted in a huge technical benefit as it does not require highly challenging and tedious steps of post-imaging tissue demarcation and particular computational selection of epithelial cells for analysis.

We conclude that tissue characterization through cell-by-cell 3D high-content analysis using Biomarkers I (DAPI, 5mC, 5hmC and AMACR) showed favorable predictive values when combined with the two types of statistical learning methods: a) the logistic model to predict composition of aberrant versus benign cells in tissue samples, and b) KNN classification to correlate cell composition with pathological categories (including PCa stages) and PCa grades, both for the detection of possible occult cancer at first and follow-up diagnoses, and the prediction of the degree of malignancy — especially the greater than GS3 component within biopsied tissue. The results encourage the validation of Biomarkers I in affiliation with 3D high-content screening and both statistical models in larger patient cohorts.

## MATERIALS AND METHODS

### Cultured cells and tissues

For the purpose of this analysis we used deidentified archived tissues from eight PCa patients and two well-characterized human cell types of prostatic origin. Cultured cells included primary human prostate epithelial cells (HPrEpiC, ScienCell, Carlsbad, CA) as normal primary cells, and LNCaP (American Type Culture Collection, Manassas, VA), an androgen-sensitive prostate cancer cell line. For this study we used HPrEpiC at an early passage to rule out a decrease in global DNA methylation due to proliferative aging in culture, which is typical for primary cells and especially for these cells, as published by Oh et al. [31]. Cells were cultured at 37°C and 5% CO_2_ following standard culture procedures as previously described in [31]. For each patient we analyzed tissues taken at different diagnostic time points, including baseline diagnosis —first biopsy (biopsy 1) and if available second biopsy (biopsy 2)— and paired prostatectomy. For each time point two needle biopsies (as displayed in Supplementary Figure 1) were labeled and analyzed. While the diagnosis and the characterization of the two cell types where known to us for the development dataset, the status on each cell was not. Therefore, we assumed that all cells coming from healthy tissues were normal and all cells coming from tumor tissues were malignant. We judged the performance of our classification methods under these assumptions.

### Immunofluorescence

Tissue sections of 10 μm thickness were deparaffinized, fixed with 4% paraformaldehyde/phosphate buffered saline, and subjected to antigen-retrieval using Target Retrieval Solution (Dako, Carpinteria, CA) according to the manufacturer's protocol, all prior to labeling procedures. Immunofluorescence labeling of cells and tissues was performed according to previously established protocols [36]. Unconjugated primary antibodies used were: monoclonal mouse anti-5-methylcytosine clone 33D3 (Aviva Systems Biology), polyclonal rabbit anti-5-hydroxymethylcytosine (Active Motif), polyclonal sheep anti-AMACR (R&D Systems), polyclonal goat anti-SAFB (Santa Cruz Biotechnology), polyclonal rabbit anti-H3K9me3 (Active Motif), monoclonal mouse anti-H3K27me3 (Active Motif), and monoclonal rat anti-AR (Santa Cruz Biotechnology). Matching secondary antibodies (all from Life Technologies, now Thermo Fisher Scientific) included: Alexa 488-conjugated donkey anti-mouse IgG (H+L), Alexa 555-conjugated donkey anti-sheep IgG (H+L); Alexa 555-conjugated donkey anti-goat IgG (H+L), Alexa 594-conjugated goat anti-rabbit IgG (H+L), Alexa 647-conjugated chicken anti-rabbit IgG (H+L), Alexa 647-conjugated donkey anti-sheep IgG (H+L), and Alexa 680-conjugated goat anti-rat IgG (H+L). The specimens were counterstained with 4′,6-diamidino-2-phenylindole (DAPI), prior to mounting in ProLong Gold (Thermo Fisher Scientific).

### Image acquisition and analysis

Confocal imaging of labeled cells and tissues was performed using a TCS SP5 X supercontinuum microscope (Leica Microsystems). This microscope essentially provides flexible settings of excitation and emission wavelengths in a continuous range between 470nm and 670nm with 1nm increment. Serial optical sections were collected at increments of 500 nm with a Plan-Apo 63×1.3 glycerol immersion lens. The pinhole size was consistently 1.0 airy unit. To avoid channel bleed through due to overlap of emission spectra, images were acquired serially: first DAPI, Alexa 555, and Alexa 647 dyes, followed by Alexa 488 and Alexa 594. The typical image size was 1576×1576 with a respective voxel size of 189 nm ×189 nm × 500 nm (x, y, and z axes), and a dynamic intensity range of 12 bits per pixel in all four channels. All biomarker signals from optical sections were recorded into separate channels. All images were acquired under nearly identical conditions and modality settings. The drift of the settings during acquisition was considered minimal and therefore neglected. 3D image analysis was performed as described in [29, 31, 38]. The resulting dat-files were incorporated into software for statistical analysis, as described in the following section.

### Exploratory statistical analysis

All analyses were conducted with STATA 14 (StataCorp., College Station, TX). To address the first aim of our study as well as in the exploratory step of our study we assessed the degree of changes (variance and covariance) of the two sets of biomarkers in correlation with tissue cancer pathology (degree of malignancy) as indicated by 1) pathological categories and 2) GS. To this end we decided to use principle component analysis, which is one of the oldest and most widely used multivariable analyses. It was originally developed by Pearson (1901) and independently by Hotelling (1933) [55]. Using PCA the goal is to explain most of the variability of the data while trying to reduce the dimensionality (number of variables) of the dataset. PCA achieves this by linear transformation of the original variables (*x_1_, x_2_,…, x_p_*) into a new set of variables (*y_1_,y_2_,…,y_p_*) called principle components, where

$$\begin{array}{ccc} y_{1} & = & a_{11}x_{1}+a_{12}x_{2}+\ldots +a_{1p}x_{p}\\ y_{2} & = & a_{21}x_{1}+a_{22}x_{2}+\ldots +a_{2p}x_{p}\\ & = & \;+\;+\;+\\ y_{p} & = & a_{P1}x_{1}+a_{P2}x_{2}+\ldots +a_{Pp}x_{p} \end{array}$$ Eqs. 1

Using principle component analysis, the newly produced variables were derived in decreasing order of importance pertaining to the amount of variation they explain of the original variables. For example, PC 1 explains for as much of the variability of the original data as possible. The PC 2 explains as much of the remaining variance as possible under assertion that it is not correlated to PC 1, etc. This reduction is useful as our data could be graphically summarized with less dimensions, instead of exploring the covariance/correlation of the pairwise relationship between the markers within each of the two marker sets assessed in this study. We used this visualization to identify outliers and grouping (clusters) of cells in respect to their pathological categories and GS. The goal was determine the set of biomarkers that provides better clustering (more clear segregation) of the groups of cells among various tissue specifications [56]. We also looked at the loading matrix (the set of eigenvectors) to find out if we can further reduce the number of markers within each biomarker set. As tissue sections were either simultaneously labeled for the four Biomarkers I (DAPI, 5mC, 5hmC, AMACR) or the five Biomarkers II (DAPI, SAFB, H3K9me3, H3K27me3, AR) —with DAPI used in both sets— it was not possible to assess on a cell-by-cell basis the combination of all eight different biomarkers.

### Statistical learning methods for diagnosis and prognosis of cancer

Two statistical analyses were considered for pursuing the objectives of our study. First, we focused on the development of a logistic model that would determine the probability of a cell being non-cancerous (normal, benign) or cancerous (malignant, transformed), based on two separate sets of Biomarkers I and Biomarkers II. We used a group of five subjects that were diagnosed with PCa at the time of biopsy or prostatectomy, as our development dataset.

The logistic analysis was performed once only with the cells located in the epithelial tissue compartment and then repeated once with all imaged cells from all tissue compartments (including the epithelial compartment). All data were analyzed in a cell-by-cell manner. In other words, for each cell the logistic model estimated a probability of being cancerous (malignant) or non-cancerous (normal). As a first step of building the multivariate logistic model, we conducted a univariate logistic regression to identify those biomarkers that were significantly associated with cancerous cells. For the purpose of developing the logistic model, we assumed that cells originating from malignant tissue are malignant cells and the cells originating in what we call normal tissue were all normal. Logistic regression is a linear regression approach used when the outcome variable *dichotomous* as we define it above.

$$E\;y=\beta_{0}+\beta_{1}x_{1}+\;+\beta_{P}x_{P}$$ Eq. 2

The aim of this analysis is to estimate the probability that the outcome takes the value of interest (malignant, normal cell) depends on the explanatory variables which in our case was the set of Biomarkers I or II (Equation 2). Nevertheless, there are two major obstacles when modeling a *dichotomous* outcome: 1) the prediction of the model must satisfy 0≤ E(y)≤1, whereas a linear predictor can yield any value from plus to minus infinity; and 2) our outcome is not normally distributed but it is rather binomially distributed. Both issues were resolved by logit transforming the left side of equation 2 where,

$$\text{logit}(E\;y)=\log\left(\frac{E\;y}{1-E\;y}\right)$$

transforming equation 2 into,

$$\text{logit}(E\;y)=\beta_{0}+\beta_{1}x_{1}+\;+\beta_{P}x_{P}$$ Eq. 3

Once the coefficients of equation 3 are estimated it is easy to estimate the probability *E[y]* using inverse logit function. Once we were able to accurately estimate the parameters of logistic model, we assessed how effectively the model describes the outcome. This is referred to as *goodness-of-fit*. To determine the “optimized” cutoff that is required to build the classification tables (Figure 6 and 7), we used summary tables of sensitivity and specificity for a given cutoff value that varied from 0-1 with increments of .05. Generally, the goal is to maximize both sensitivity and specificity by varying the cutoff point [51]. We also used AUC of the ROC curve. The AUC of ROC curve, which ranges from 0 to 1, provides a measure of the models ability to discriminate between malignant and non-malignant cells. In order to interpret the ROC we adopted the general rules as suggested by Hosmer and Lemeshaw [51]:

If ROC=0.5 no discrimination (the model is not better than flipping a coin)

If ROC>0.7 and ROC≤0.8 acceptable discrimination

If ROC>0.8 and ROC≤0.9 excellent discrimination

If ROC>0.9 and ROC≤1 outstanding discrimination

Since our models were based on cell-by-cell data, and the diagnosis was associated with the tissue, we needed to bridge the gap. An *a priori* decision was made that the largest portion of cells in each tissue should be considered as the determinant of the characteristic of that tissue as a whole, and therefore be concordant with the known diagnosis. For example, 80% of normal cells indicated that there is 80% probability that the tissue was normal and 20% probability of malignancy. This assumption had to be established because there was no conceivable way for us to assess the true state of the cells with respect to malignancy. Once we were assured that we had obtained the best logistic model given the data, we proceeded to validate the model in an independent set of five samples. Validation was necessary because a logistic model may be heavily biased by cells originating from an outlier individual [57]. For this purpose we developed an intricate validation procedure. The validation data set was comprised of: a) the two cell lines b) Patients 6, 8 and 9 and c) two prostatectomy tissue samples isolated from areas distant from the tumor that had normal appearance based on H&E staining (per expert pathological diagnosis) from Patient 5 and separately from another patient (Patient Z). The cultured cells are well established and were used as surrogates for normal and cancer tissue. We felt that while they provided an initial good assessment of our logistic model, they may not be an absolute replacement for patient tissue. Therefore, we proceeded with the analysis of three patients which were not included in the model (Patients 6, 7, and 8). While we knew the complete pathological history of Patient 6, we only knew the baseline diagnosis for patients 7 and 8 as we were blinded to their prostatectomy results. With Patient 6 we validated the logistic model predictions (also the KNN analysis) in comparison with the clinical diagnosis of this subject. Using data of patients 7 and 8 we evaluate the prognostic power of the model. Finally the normal tissue from two patients was used to assess whether the logistic model is capable of assigning probability to this tissue that may indicate that these subjects are normal or have malignancy.

Second and final, we performed two k-nearest neighbor (KNN) classifiers that would predict the two types of classifications of cells. KNN is a memory-based classifier and a model free approach [58]. We found *k* training points where *x_r_,r=1,…,k* closest in distance to *x_0_*. Despite its simplicity, KNN classification has been very successful in a large number of applications that originally faced classification challenges, such as satellite image scenes and EKG [42]. The size of the cluster of nearest neighbors (*k* parameter) for the KNN classification was determined using the training data thereby maximizing the likelihood of correct classification [58]. We determined that the best results were obtained with *k* = 5. Thus, *k* was sufficiently large to diminish noise effects in the data, yet small enough to reduce computational expenses. Instead of Euclidian distance between the neighbors, we used Mahalanobis distance [59]. As a distance measure we applied Mahalanobis transformation. Therefore, the scale of distance measure between a point *x_0_* and *x_r_* was the standard deviation. The first classification was based on baseline diagnoses (biopsies) and prostatectomy. The second version was considering GS (cancer grade) of the same specimens as an indicator of disease progression and cancer aggressiveness. The KNN classifications were developed using same development and validation datasets as for the logistic regression model. Hence analogously tissues were classified based on the category of the largest portion of cells.

### Supplementary materials

The following additional figure and tables are available in Supplementary Materials. Supplementary Figure 1 shows consecutive sample prostate tissue stained with either H&E or by immunofluorescence. Supplementary Table 1 displays the loading matrix of Biomarkers I —comprising the principal components (eigenvectors)— for the epithelial compartment only. Supplementary Table 2 shows the loading matrix of Biomarkers II, also for the epithelial compartment only.

## SUPPLEMENTARY MATERIALS FIGURES AND TABLES


