# Supplementary material for: Prostate cancer diagnosis using epigenetic biomarkers, 3D high-content imaging and probabilistic cell-by-cell classifiers
Source: Oncotarget. 2017 Jul 5;8(34):57278–301. doi: 10.18632/oncotarget.18985 (PMC5593641; doi:10.18632/oncotarget.18985)
Supplement: Supplementary file 1 [file oncotarget-08-57278-s001.pdf]

Prostate cancer diagnosis using epigenetic biomarkers, 3D high-content imaging and probabilistic cell-by-cell classifiers

SUPPLEMENTARY MATERIALS

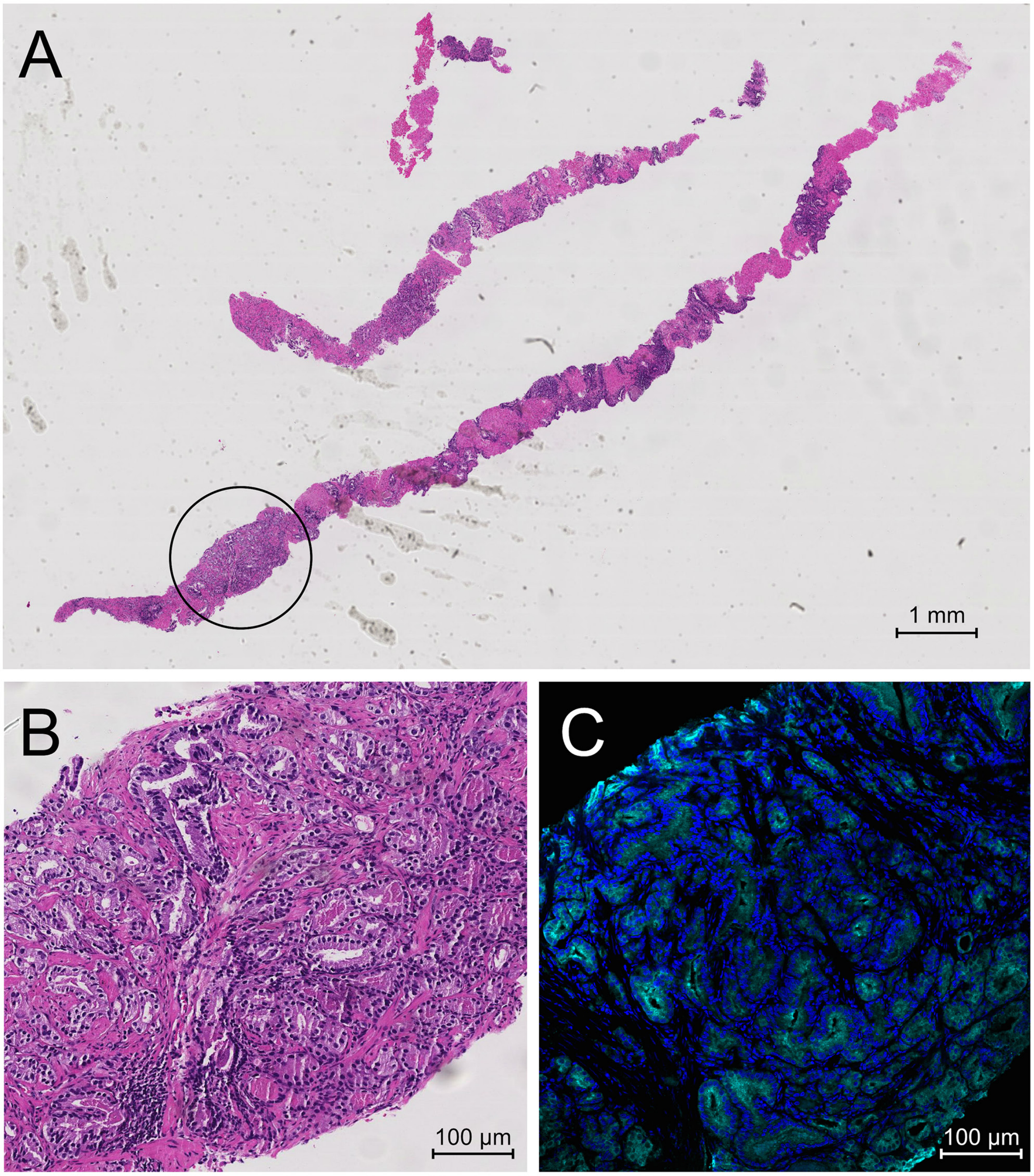

Supplementary Figure 1: Sample prostate tissue staining. (A) H&E stained needle biopsy 2 of Patient 1 showing adenocarcinoma, imaged using an automated scanner (Aperio ScanScope AT turbo, Leica Biosystems) with a 20xNA0.7 objective. Tumor compartments can be easily identified on the aerial (whole) image. **(B)** A tumor subregion (circle in Figure 2A) was 10X zoomed in to identify tumor areas for fluorescence confocal imaging and the analysis of these areas in consecutive tissue sections that were labeled by immunofluorescence. **(C)** Confocal dual-color image (mid section) of the same subregion at 40X magnification (imaged with a TCS SP5 X Supercontinuum microscope, Leica Microsystems); cell nuclei are delineated by DAPI (false-colored blue) and tumor regions highlighted by cytoplasmic AMACR (cyan).

Supplementary Table 1: Principal components (eigenvectors) = loading matrix of Biomarkers I for epithelial cells only

Variable	Comp1	Comp2	Unexplained
**DAPI**	-0.23	0.96	0.01
**AMACR**	0.60	0.13	0.20
**5mC**	0.42	0.23	0.57
**5hmC**	0.64	0.08	0.12

Supplementary Table 2: Principal components (eigenvectors) = Loading Matrix of Biomarkers II for epithelial cells only

Variable	Comp1	Comp2	Unexplained
**DAPI**	0.15	0.75	0.32
**nAR**	0.56	0.22	0.11
**SAFB**	0.46	`-0.48	0.18
**H3K9me3**	0.33	0.33	0.59
**H3K27me3**	0.58	-0.22	0.06
